# Different Dynamic Distribution in Chickens and Ducks of the Hypervirulent, Novel Genotype Fowl Adenovirus Serotype 4 Recently Emerged in China

**DOI:** 10.3389/fmicb.2017.01005

**Published:** 2017-06-06

**Authors:** Qing Pan, Yanchao Yang, Zhibin Shi, Linlin Liu, Yulong Gao, Xiaole Qi, Changjun Liu, Yanping Zhang, Hongyu Cui, Xiaomei Wang

**Affiliations:** ^1^Division of Aivan Infectious Diseases, State Key Laboratory of Veterinary Biotechnology, Harbin Veterinary Research Institute of Chinese Academy of Agricultural SciencesHarbin, China; ^2^Jiangsu Co-innovation Center for Prevention and Control of Important Animal Infectious Disease and ZoonosesYangzhou, China

**Keywords:** novel FAdV-4, duck origin, dynamic distribution, real-time PCR, different hosts

## Abstract

A hypervirulent fowl adenovirus serotype 4 (FAdV-4) has caused hepatitis-hydropericardium syndrome (HHS) with mortalities that range from 30 to 80% in outbreaks across China since 2015. The FAdV-4 strain was characterized as a novel genotype based on the specific genome characteristics. However, our understanding of the dynamic distribution, tissue tropism, and pathogenesis of the novel FAdV-4 is incomplete. In this study, a new, sensitive and FAdV-4-specific real-time PCR was developed and applied to detect the dynamic distribution of the duck origin, novel FAdV-4 strain HLJDAd15 in experimentally infected special-pathogen free (SPF) chickens and ducks. Notably, the pathogenicity and replication pattern of HLJDAd15 were completely different between chickens and ducks. Severe hydropericardium and 10% mortality were induced in chickens, whereas no clinical signs were observed in any duck. The virus replicated was detected throughout the study in both chickens and ducks. However, only one replication peak with a high virus concentration appeared in chickens at 5 days post infection (dpi), whereas two peaks with relatively low virus titres appeared in ducks at 7 and 21 dpi. Thus, ducks could be a natural reservoir of the novel FAdV-4 absent of clinical signs, and a new transmission route from ducks shedding FAdV-4 continually to chickens was revealed, which might aggravate the outbreak of HHS in chickens. This study provides the first accurate quantitative data for the replication kinetics of the novel FAdV-4 in different hosts. The different pathogenicity, dynamic distribution and replication pattern in chickens and ducks provide a foundation for further clarification of the pathogenesis of the novel FAdV-4.

## Introduction

Adenoviruses, family *Adenoviridae*, are non-enveloped and double-stranded DNA viruses (Stewart et al., 1993). The International Committee on Taxonomy of Viruses (Harrach and Kaján, 2011) separates the Adenoviridae into five genera: *Mastadenovirus, Aviadenovirus, Siadenovirus, Atadenovirus*, and *Ichtadenovirus*. Fowl adenoviruses, members of the genus *Aviadenovirus*, are further separated into either five species (designated FAdV-A to FAdV-E) based largely on molecular criteria and restriction enzyme digest pattern or twelve serotypes (designated FAdV-1 to 8a and -8b to 11) based on the results of serum cross-neutralization tests (Hess, 2000). The FAdVs are associated with several notable diseases in chickens and other birds, such as inclusion body hepatitis (IBH), hepatitis-hydropericardium syndrome (HHS), and gizzard erosion (GE) (Domanska-Blicharz et al., 2011; Marek et al., 2012).

Hydropericardium-hepatitis syndrome was first reported in Pakistan in 1987 (Khawaja et al., 1988), and subsequent outbreaks have also been reported worldwide in recent years, including in America, Mexico, Hungary, Poland, Peru, Ecuador, Chile, Japan, Korea, India, and China, resulting in significant economic losses to the poultry industry (Hess et al., 1999; Griffin and Nagy, 2011; Grgić et al., 2013; Kaján et al., 2013; Niczyporuk, 2016). HHS appears with high frequency in chickens, particularly in broilers, with a mortality that ranges from 10 to 100% (Vera-Hernández et al., 2016; Pan et al., in press). However, the disease has rarely emerged in other hosts with severe clinical signs and high mortality. Fowl adenovirus serotype 4 (FAdV-4) plays a primary role in the etiology of HHS based on epidemiological studies, and many hypervirulent strains have been isolated from cases of HHS (Vera-Hernández et al., 2016; Ye et al., 2016; Pan et al., in press). For diagnosis of FAdVs infection, several routine PCR methods have been reported based on serotype-specific hexon gene (Steer et al., 2009; Kaján et al., 2013) and a SYBR Green based real-time PCR within 52K gene was developed for detection and quantitation of all FAdV species (Günes et al., 2012). However, a sensitive serotype 4 specific quantitate PCR has not been available and urgently needed for diagnosis and deeper investigation of FAdV-4, especially the emerged novel FAdV-4.

Since June 2015, severe outbreaks of HHS with high mortalities from 30 to 100% have emerged in several different areas of China. Many hypervirulent FAdV-4 strains have been isolated and identified from different cases, and a reproduction experiment revealed high mortality in SPF chickens (Zhao et al., 2015; Li H. et al., 2016; Niu et al., 2016). Given the complete genome sequence analysis, Chinese hypervirulent FAdV-4 strains show many new characters, such as the natural large deletions of ORF19 and ORF27, and are identified as a novel genotype (Li L. et al., 2016; Liu et al., 2016; Ye et al., 2016). Although many studies on chicken origin FAdV-4 have been conducted, less is known about the duck origin FAdV-4 (Chen et al., 2016). In this study, a new, FAdV-4-specific real-time PCR was developed and applied to detect the dynamic distribution of a duck origin FAdV-4 in experimentally infected SPF chickens and ducks, which revealed different replication patterns of the novel FAdV-4 between chickens and ducks, helping to further understand the pathogenicity and transmission mechanism between different hosts.

## Materials and Methods

### Ethics Statement

The animal experiments with chickens and ducks were approved by the Animal Care and Use Committee of Harbin Veterinary Research Institute (Harbin, China) and performed in accordance with the ‘Guidelines for Experimental Animals’ of the Ministry of Science and Technology (Beijing, China). All SPF chickens and ducks were cared for in accordance with humane procedures.

### Virus

Duck origin FAdV-4 (HLJDAd15) was isolated from the field in 2015 in Heilongjiang Province, China (Pan et al., in press), and is a novel genotype of FAdV-4 based on the complete genome sequence (GenBank No. KX538980).

### Animal Infection

Forty of each 1-day-old specific pathogen free (SPF) chickens and ducks were purchased from the Experimental Animal Center of Harbin Veterinary Research Institute (HVRI) of the Chinese Academy of Agricultural Sciences (CAAS), China. Thirty SPF chickens and ducks were orally inoculated with 10^3.0^ ELD_50_ doses of the homogenates of HLJDAd15 in 0.2 ml of phosphate-buffered saline (PBS) at 35 days old, whereas the 10 other SPF chickens and ducks served as negative controls. The challenged and control groups were separately housed in different negative-pressure isolators and monitored daily for 30 days.

### Sample Collection

At 1, 3, 5, 7, 10, 14, 21, 28, 35, and 42 days post infection (dpi), three infected birds and one control bird were sacrificed by intraperitoneal injection of sodium pentobarbital. Tissue samples of heart, liver, spleen, lung, kidney, thymus, bursa, caecal tonsi, proventriculus, and rectum were collected. The homogenates of 10 to 100 mg of tissue samples in 200 μl of PBS were subjected to DNA extraction or stored at -80°C. Three serum samples were also collected at each dpi and stored at -80°C until used for antibody assays.

### Viral DNA Extraction

The viral DNA of tissue samples was extracted from the tissue homogenates using a DNeasy Tissue kit (Qiagen, Hilden, Germany) according to the manufacturer’s instructions, whereas the positive DNA or proviral DNA of FAdV-1, Marek’s disease virus (MDV) (Lv et al., 2016), chicken infectious anaemia virus (CAV) (Wang et al., 2017), avian gyrovirus 2 (AGV2) (Yao et al., 2017), reticuloendotheliosis virus (REV) (Jiang et al., 2013), and avian leukosis virus subgroup A, B, and J (ALV-A, ALV-B, ALV-J) (Gao et al., 2014) were maintained in our laboratory.

### Routine PCR

The primers used in this study to detect the genotype of FAdV were designed based on the L1 region of the Hexon gene (FAd-F: 5′-AACTTCGACCCCATGTCGCGTCAGG-3′ and FAd-R: 5′-TGGCGAAAGGCGTACGGAAGTAAGC-3′). The PCR products were subjected to electrophoresis in a 1% (w/v) agarose gel. The PCR procedure consisted of an initial incubation for 5 min at 95°C; 35 cycles of 30 s at 95°C, annealing for 60 s at 55°C, and extension for 60 s at 72°C; and a final extension for 5 min at 72°C.

### Real-Time PCR

Real-time PCR was performed with a LightCycler 480 real-time thermocycler (Roche Instrument Center, Switzerland). The primers were designed based on the FAdV-4 specific L1 region of the Hexon gene (Ganesh et al., 2001; Steer et al., 2009; Park et al., 2017) as follow: forward primer 5′-CAGTTCATTTCCGCCACC-3′ and reverse primer 5′-GCAGCCGTTGAGCCTTTT-3′. The relative TaqMan probe was a 23 bp oligonucleotide, 5′FAM-TCTGTCGTGACATTTCGGGTGGG-3′TAMRA, which was labeled with FAM at the 5′ end and TAMRA at the 3′ end. The reactions were conducted with a predenaturation step at 95°C for 5 min and amplification for 40 cycles at a melting temperature of 95°C for 10 s and an elongation at 65°C for 40 s (Qin et al., 2013; Gaudreault et al., 2015). The fluorescent signal was collected during the elongation step. A 341 bp fragment of the L1 region of Hexon was cloned into the pEASY-T1 vector (TransGen, Beijing, China) to prepare a standard recombinant plasmid pAD341. Serial dilutions of the standard plasmid from 1 × 10^1^ to 1 × 10^11^ copies/μl were used to produce a standard curve.

### Histopathology

Tissue samples were fixed in 10% formalin for 48 h at room temperature (RT), and then routinely processed, embedded in paraffin wax, and cut into 5-μm sections. The sections were stained with hematoxylin and eosin (HE) and examined using light microscopy.

### Antibody Production

Antisera against the viral isolate HLJFAd15 were collected from 5-week-old SPF chickens at 1, 3, 5, 7, 10, 14, 21, 28, 35, and 42 days post infection. Serum samples were tested for FAdV-specific antibodies using a commercial enzyme-linked immunosorbent assay (ELISA) kit (BioChek, Scarborough, ME, United States).

## Results

### Development of Real-Time PCR

**FIGURE 1:**
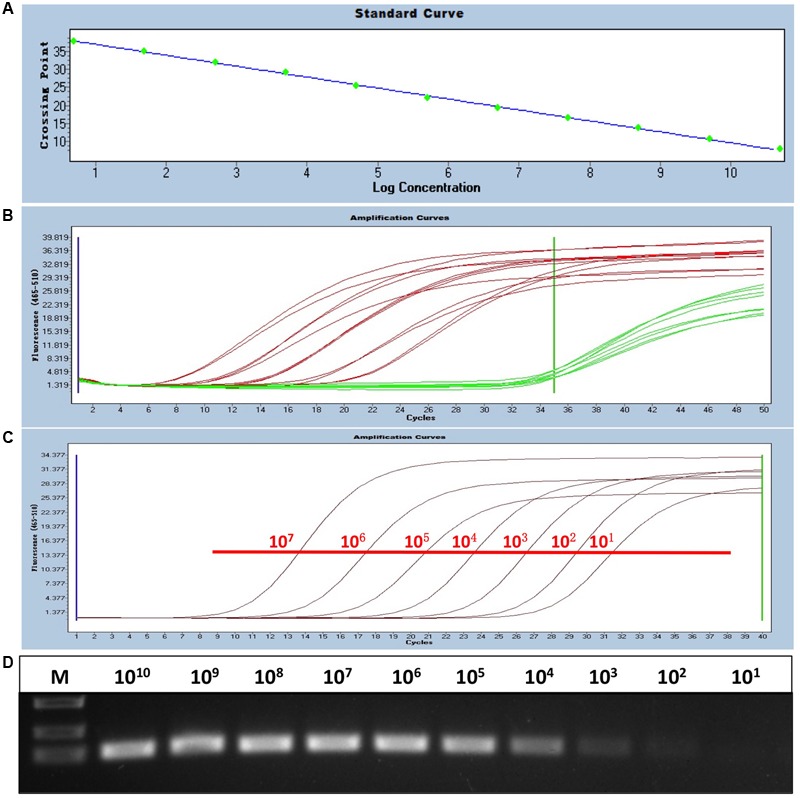
Establishment of the sensitive, FAdV-4-specific real-time PCR. **(A)** Standard curve of the real-time PCR. The standard plasmid was serially diluted from 1 × 10^1^ to 1 × 10^11^ copies/μl, and a good correlation (error = 0.085, efficiency = 2.152) between CT values and copy numbers was produced. **(B)** Specificity of the real-time PCR for FAdV-4. Red curves (HLJDAd15 and 11 other FAdV-4 isolates identified by routine PCR) were considered positive, and green curves (FAdV-1, MDV, CAV, AGV2, REV, ALV-A, ALV-B, and ALV-J) were considered negative. **(C,D)** Sensitivity of the real-time PCR. The detection limit of the real-time PCR (10^1^ copies/μl) was at least 10-fold higher than that of routine PCR (above 10^2^ copies/μl).

To generate a standard curve for the real-time PCR, the standard recombinant plasmid pAD341 was serially diluted from 1 × 10^1^ to 1 × 10^11^ copies/μl, and the serial-diluted plasmids were detected by the real-time PCR assay. Threshold cycle values (*C*_T_) were plotted against the known copy numbers of the standard controls. The good correlation (error = 0.085, efficiency = 2.152) between CT values and copy numbers is shown in **Figure 1A**.

### Specificity of Real-Time PCR

Viral DNA from 11 chicken origin and 1 duck origin FAdV-4 isolates was subjected to the established real-time PCR and was well detected, whereas fluorescent signals from viral DNA of FAdV-1, MDV, CAV, and AGV2 and proviral DNA of REV, ALV-A, ALV-B, and ALV-J were not detected, indicating the specificity of the real-time PCR for FAdV-4 (**Figure 1B**).

### Sensitivity of Real-Time PCR

The sensitivity of the real-time PCR was compared with that of routine PCR. The recombinant plasmid pAD341 was used as a template. The results showed that the detection limit of the real-time PCR established in this study was fewer than 10^1^ copies/μl (**Figure 1C**), whereas the detection limit of routine PCR was more than 10^2^ copies/μl (**Figure 1D**), demonstrating that the sensitivity of our real-time PCR was at least 10-fold higher than that of routine PCR.

### Dynamic Distribution in SPF Chickens of HLJDAd15

**FIGURE 2:**
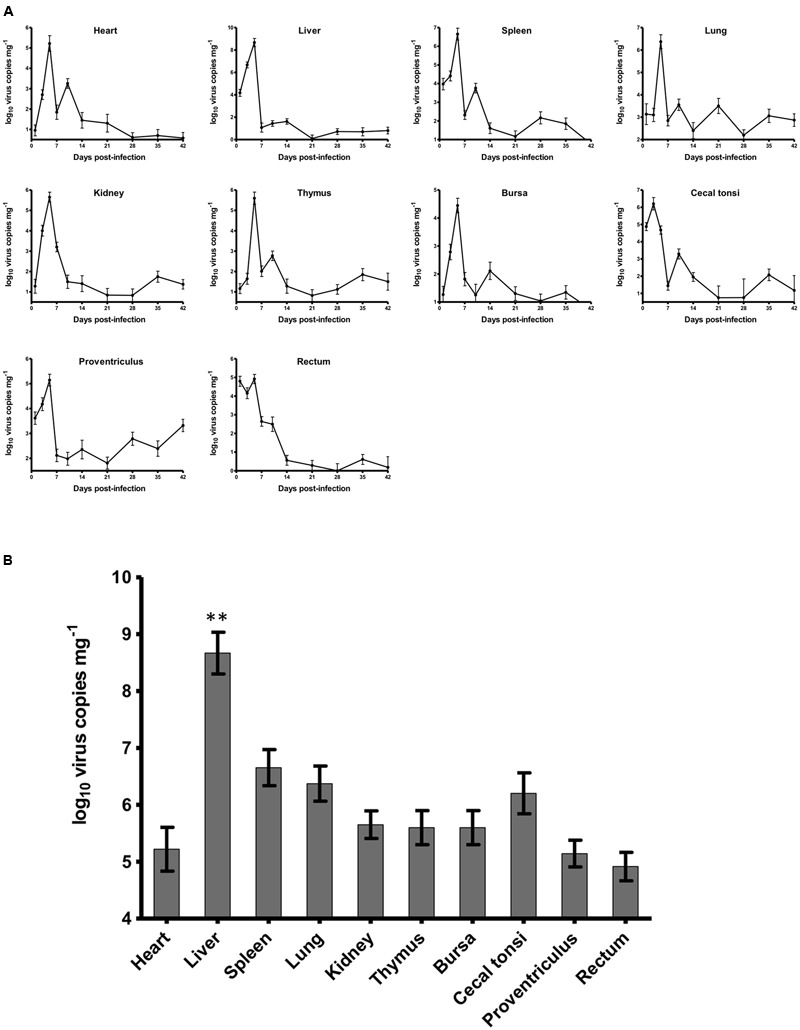
Dynamic distribution of the novel FAdV-4 in experimentally infected SPF chickens. **(A)** One replication peak occurred in the heart, liver, spleen, lung, kidney, thymus, bursa, proventriculus, and rectum at 5 dpi and in caecal tonsi at 3 dpi. **(B)** The peak concentration of virus replication in the liver was significantly higher than that in other tissues (^∗∗^*p* < 0.01).

For chickens in the experimentally infected group, 1 chicken and 2 chickens died on 3 and 5 dpi, respectively. The dead chickens showed severe hydropericardium and IBH. The dynamic distributions of the duck origin FAdV-4 in different tissues of SPF chickens were detected by real-time PCR (**Figure 2A**). The virus DNA was detectable throughout the experiment, and the peak of virus replication appeared at 5 dpi in all tissues except in caecal tonsi with the peak at 3 dpi. After 14 dpi, the concentration of virus in all tissues decreased and remained at a low level without another replication peak until 42 dpi at the end of the experiment. The highest number of virus copies in different tissues was compared among the heart, liver, spleen, lung, kidney, thymus, bursa, proventriculus, and rectum at 5 dpi and in the caecal tonsi at 3 dpi (**Figure 2B**). The virus loads in all tissues were higher than 10^5^ copies mg^-1^, with the concentration in livers significantly higher (*p* < 0.01) than that in any other tissue. All chickens in the control group were alive and healthy at the end of the experiment, and viral DNA was not detected by either routine PCR or the real-time PCR.

### Histopathology in Different Tissues

**FIGURE 3:**
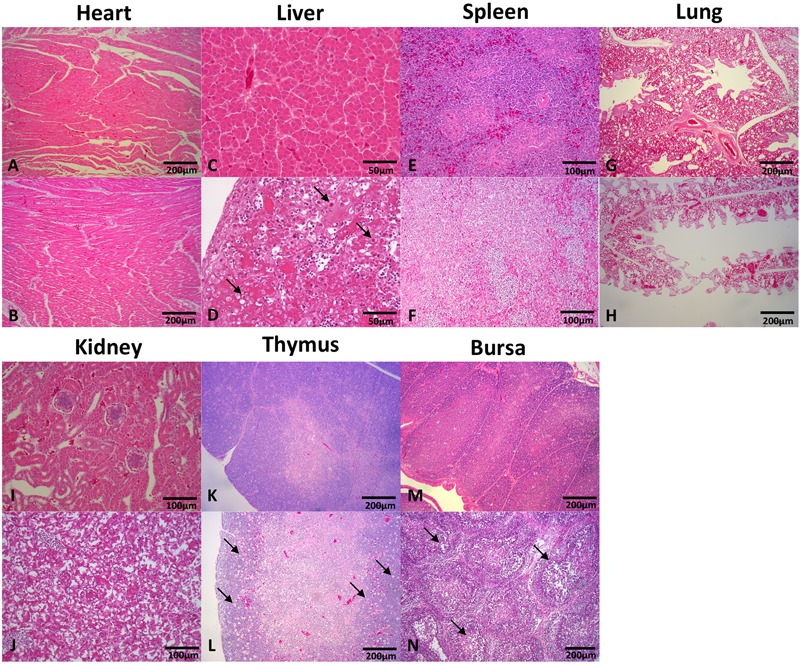
Histopathology in tissues from dead chickens inoculated with HLJDAd15. Tissues in infection and control groups were collected, fixed, cut into sections, and stained with HE. Generally, there was no significant histopathology damages presented in the heart **(A)**, liver **(C)**, spleen **(E)**, lung **(G)**, kidney **(I)**, thymus **(K)**, and bursa **(M)** of chickens in control group, while massive pathological damages were observed in various tissues of chickens in infection group: **(D)** degeneration, vacuolar necrosis and basophilic inclusion bodies presented in liver cells; **(J)** large numbers of vacuolar necrosis showed in kidney cells; **(L)** large numbers of macrophages proliferation and cortical lymphocyte depletion appeared in thymus; severe reduction and necrosis of lymphocytes showed in spleen **(F)** and bursa **(N)**; no significant differences were observed in heart **(B)** and lung **(H)** compared with the control group.

Tissues of the heart, liver, spleen, kidney, thymus, and bursa of chickens in different groups were collected 5 days post challenge and fixed, cut into sections, and stained with HE (**Figure 3**). Generally, there was no significant histopathology damages presented in chickens of control group, while massive pathological damages were observed in various tissues of chickens in infection group: degeneration, vacuolar necrosis and basophilic inclusion bodies presented in liver cells (**Figure 3D**); large numbers of vacuolar necrosis showed in kidney cells (**Figure 3J**); large numbers of macrophages proliferation and cortical lymphocyte depletion appeared in thymus (**Figure 3L**); severe reduction and necrosis of lymphocytes showed in spleen (**Figure 3F**) and bursa (**Figure 3N**).

### Dynamic Distribution in SPF Ducks of HLJDAd15

**FIGURE 4:**
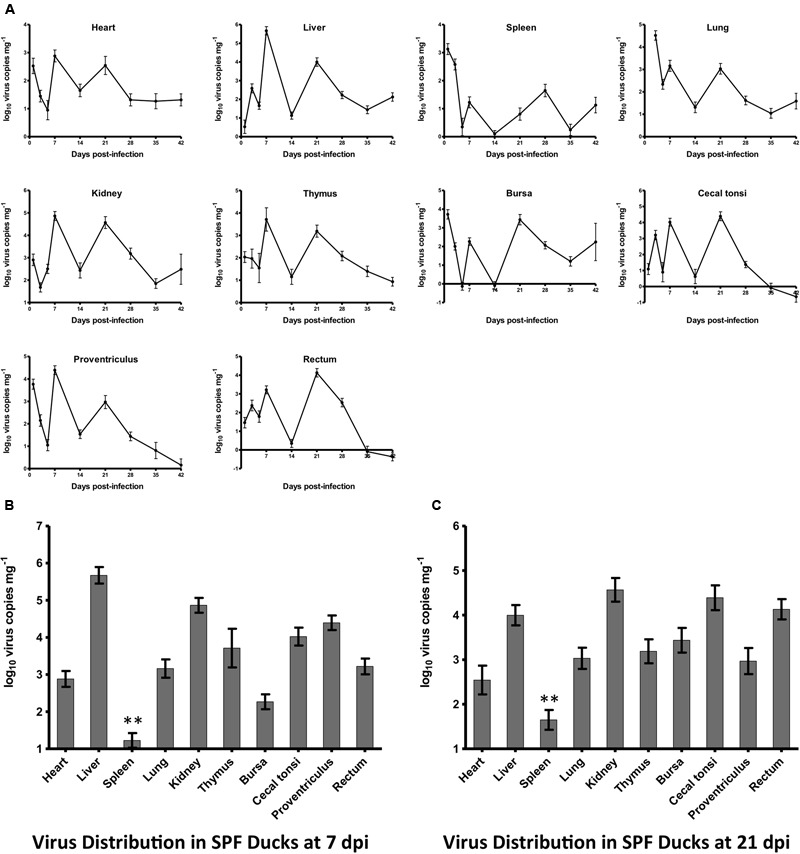
Dynamic distribution of the novel FAdV-4 in experimentally infected SPF ducks. **(A)** Double replication peaks occurred in the heart, liver, spleen, lung, kidney, thymus, bursa, caecal tonsi, proventriculus, and rectum at 7 and 21 dpi. The peak concentrations of virus replication at 7 dpi **(B)** and 21 dpi **(C)** were compared, and the virus load in the spleen at both times was significantly lower than that in other tissues (^∗∗^*p* < 0.01).

All ducks in both experimentally infected and control groups remained alive and did not show visible clinical signs by 42 dpi at the end of the experiment. The dynamic changes of HLJDAd15 in different tissues of SPF ducks were detected by real-time PCR (**Figure 4A**). The virus DNA was detectable throughout the experiment, and two peaks of virus replication appeared at 7 and 21 dpi (28 dpi for spleen). The virus copy peaks at both 7 dpi (**Figure 4B**) and 21 dpi (**Figure 4C**) in the heart, liver, spleen, lung, kidney, thymus, bursa, proventriculus, rectum and caecal tonsi were analyzed. The results showed that the virus concentrations in all tissues were between 10^2^ and 10^5^ copies mg^-1^ tissue, with the exceptions of concentrations higher than 10^5^ in livers at 7 dpi and lower than 10^2^ in spleens from 5 dpi. Furthermore, the trends between the two virus replication peaks in the different tissues were similar.

### Comparison of Virus Concentrations between Chickens and Ducks

**FIGURE 5:**
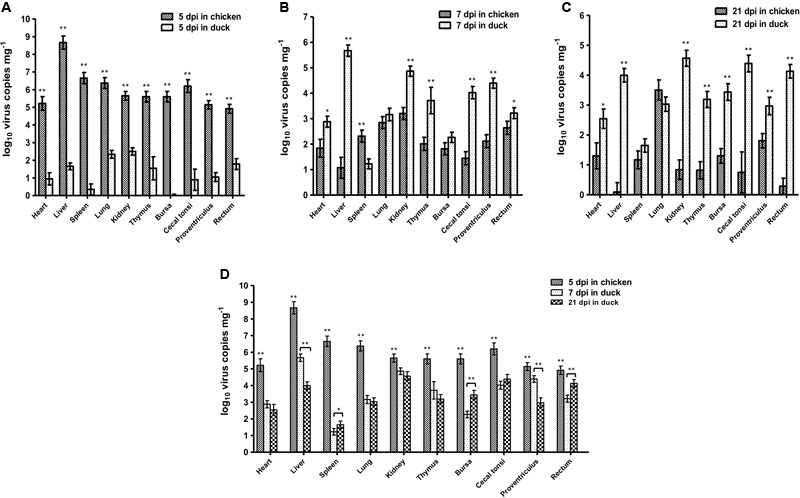
Comparison of Virus Peak Concentration between Chickens and Ducks. The peak concentrations of duck origin FAdV-4 showed at 5 dpi in SPF chickens and 7 and 21 dpi in SPF ducks. The comparisons of virus titres between chickens and ducks at 5 dpi **(A)**, 7 dpi **(B)**, and 21 dpi **(C)** were separately analyzed. Furthermore, the highest virus titres in chickens at 5 dpi and in ducks at 7 and 21 dpi were compared **(D)**. The peak concentrations of FAdV-4 in all tissues of chickens were significantly higher (^∗∗^*p* < 0.01) than those in ducks. The highest concentration was in the livers of chickens (^∗∗^*p* < 0.01), whereas the lowest concentration of virus copies appeared in the spleens of ducks (^∗∗^*p* < 0.01).

Based on the dynamic distributions of the duck origin FAdV-4, the virus replications in different tissues of SPF chickens and ducks were compared at 5 dpi (**Figure 5A**), 7 dpi (**Figure 5B**), and 21 dpi (**Figure 5C**), respectively. The results showed that the virus copies in different tissues of chickens were significantly higher (*p* < 0.01) than ducks at 5 dpi, when the unique replication peak showed in chickens. However, the virus concentrations in chickens rapidly decreased after 5 dpi and lower than ducks at 21 dpi, when the second replication peak appeared in ducks. Moreover, the peak concentrations in different tissues of different hosts were also analyzed (**Figure 5D**). The data showed that the virus peak concentrations in all tissues of chickens were significantly higher (*p* < 0.01) than those at both peaks in ducks, and particularly in the liver, heart, spleen, lung, thymus, bursa, and caecal tonsi, the virus copies were 100-fold higher in chickens than in ducks. Notably, the highest concentration of more than 10^8^ copies mg^-1^ in livers was significantly higher (*p* < 0.01) than that in any other tissues in chickens and ducks at any other times, and the concentration of virus copies in the spleen of ducks was significantly lower (*p* < 0.01) than that in other tissues of ducks.

### Antibody Production

**FIGURE 6:**
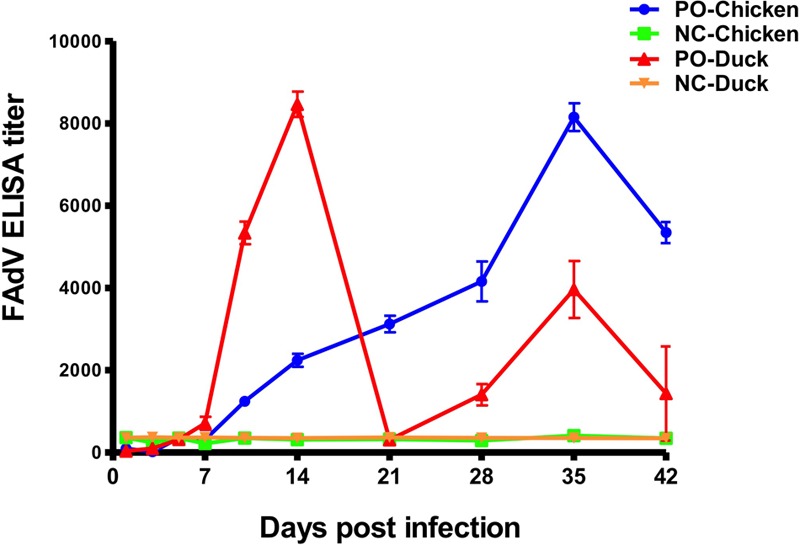
Antibody responses of experimentally infected SPF chickens and ducks. The dynamic curves of antibody response showed only one peak in chickens (Blue curve) but two peaks in ducks (Red curve), and the peak in chickens occurred later than the early peak of ducks and approximately at the same time as the late peak in ducks. All birds in the control groups showed negative antibody responses.

An antibody response of SPF chickens and ducks against FAdV was detected at 10 different times (**Figure 6**) post infection. The FAdV-specific antibody response was negative in all control birds at all times. From 7 dpi, the antibody titres of chickens continued to increase until reaching a peak at 35 dpi and then began to decrease to 42 dpi. The antibody titres of ducks were completely different from those of chickens and increased from 7 dpi, with the first peak 3 weeks earlier than that of chickens at 14 dpi and the second one at 35 dpi. The peak antibody titre was almost equal between chickens and ducks.

## Discussion

The HHS associated with FAdV-4 infection has emerged since 2015 and is reported in different provinces of China, inducing huge economic losses for poultry production (Li H. et al., 2016; Niu et al., 2016). The FAdV-4 that caused a recent epidemic in China was isolated and characterized as a novel genotype of FAdV-4 (Liu et al., 2016; Ye et al., 2016). Although many studies have been conducted on virus isolation, characterization and pathogenicity to chickens of the novel FAdV-4, less is known about the replicator dynamics *in vivo*, which is critical for enclosing the pathogenesis of the emerged novel FAdV-4. As previous reported, Several routine PCR methods have been reported based on hexon gene (Steer et al., 2009; Kaján et al., 2013) and a SYBR Green based real-time PCR within 52K gene was developed for detection and quantitation of all FAdV species (Günes et al., 2012). However, a sensitive serotype 4 specific quantitate PCR has not been available and urgently needed for diagnosis and deeper investigation of the emerged novel FAdV-4. In this study, a novel TaqMan-based, FAdV-4-specific quantitative real-time PCR within serotype-specific hexon gene was developed and applied to detect the dynamic distribution of the novel FAdV-4 not only in chickens but also in ducks. The real-time PCR showed high specificity and sensitivity for FAdV-4, providing a helpful tool for intensive study of the emerged, novel virus.

Chickens were usually considered to the most susceptible natural hosts, including broilers, layers, breeder broilers, but some other hosts were also the potential infection hosts for FAdVs, such as falcons have been reported as the natural host and primary reservoir for FAdV, although the FAdV was characterized as a novel species (Oaks et al., 2005; Schrenzel et al., 2005). The novel FAdV-4 recently emerged in China is hypervirulent to chickens (Niu et al., 2016), particularly broilers, and can be transmitted from chicken to chicken. However, less is reported on the pathogenicity of the novel FAdV-4 to ducks, with the exception that an intracerebral injection induces a mortality of 15% (Chen et al., 2016), although the infection route was not one of natural transmission. Furthermore, the transmission mechanism of novel FAdV-4 between different hosts is poorly understood. Therefore, we chose a duck origin strain HLJDAd15 to evaluate the pathogenicity and potential transmission route of the novel FAdV-4. The results of our study showed that the duck origin FAdV-4 induced severe HHS and a mortality of 10% to chickens, whereas no visible clinical signs and mortality were found in ducks. Moreover, the replication patterns in chickens and ducks were completely different. The virus persisted throughout the experiment in both chickens and ducks, but only one stronger replication peak was detected in chickens, whereas two mildly peaks were observed in ducks. Given the continuous virus shedding occurred in the infected ducks without visible clinical signs, ducks might be an intermediate host or natural virus reservoir of the novel FAdV-4. Thus, a new transmission mechanism from ducks to chickens was revealed, which might aggravate the outbreak of HHS in chickens. Furthermore, the antibody titres were highly negatively correlated with the virus replication, which suggested that the antibody might play a critical role in inhibiting the proliferation of the virus *in vivo* and might be a key index for evaluation of vaccine development; however, the detailed functions of the antibody in virus infection require further investigation.

## Conclusion

Hepatitis-hydropericardium syndrome has been caused by outbreaks of a novel genotype FAdV-4 across China since June 2015, but little is known about this novel FAdV-4. In this study, a new, sensitive and FAdV-4-specific real-time PCR was developed and applied to detect the dynamic distribution of a duck origin FAdV-4 in chickens and ducks. The novel virus showed completely different replication patterns and antibody response in different hosts, which provide a foundation for further clarification of the pathogenesis and the epidemic transmission mechanism of the novel FAdV-4.

## Author Contributions

XW and HC conceived and designed the experiments. QP, YY, ZS, and LL performed the experiments. QP and YY analyzed the data. CL and YZ contributed reagents/materials/analysis tools. QP wrote the paper. XQ and YG were involved in the interpretation of the results and critically read the manuscript.

## Conflict of Interest Statement

The authors declare that the research was conducted in the absence of any commercial or financial relationships that could be construed as a potential conflict of interest.
